# Supplementary material for: Value of the Lung Immune Prognostic Index in Patients with Non-Small Cell Lung Cancer Initiating First-Line Atezolizumab Combination Therapy: Subgroup Analysis of the IMPOWER150 Trial
Source: Cancers (Basel). 2021 Mar 9;13(5):1176. doi: 10.3390/cancers13051176 (PMC7967121; doi:10.3390/cancers13051176)
Supplement: Supplementary file 1 [file cancers-13-01176-s001.pdf]

cancers

MDPI

Supplementary Material: Value of the Lung Immune Prognostic Index in Patients with Non-Small Cell Lung Cancer Initiating First-Line Atezolizumab Combination Therapy: Subgroup Analysis of the IMPOWER150 Trial

Ashley M. Hopkins, Ganessan Kichenadasse, Ahmad Y. Abuhelwa, Ross A. McKinnon, Andrew Rowland and Michael J. Sorich

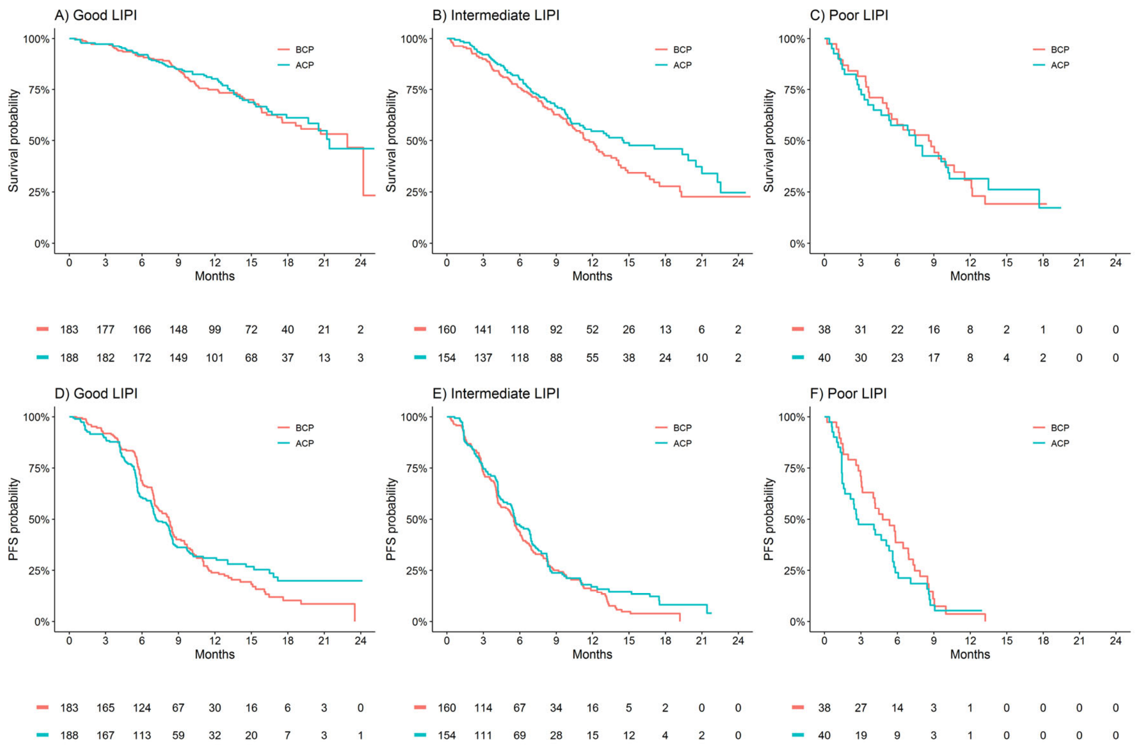

Figure S1. Kaplan Meier estimates of overall survival and progression-free survival in the randomized arms of IMpower150 (ACP versus BCP), subgrouped by LIPI.

Table S1. Summary of patient characteristics by study arm.

**Patient Characteristics**	**BCP No. 400**	**ACP No. 402**	**ABCP No. 400**	***P***
Evaluable pts	381	382	385	
Age (years)	63 (57–69)	63 (57–68)	63 (57–70)	0.54
Sex				1
Male	239 (60%)	241 (60%)	240 (60%)	
Female	161 (40%)	161 (40%)	160 (40%)	
ECOG PS				0.28
0	179 (45%)	180 (45%)	159 (40%)	
1	218 (55%)	222 (55%)	238 (60%)	
Missing	3 (1%)	0 (0%)	3 (1%)	
Race				0.77
White	335 (84%)	331 (82%)	322 (80%)	

Asian	46 (12%)	48 (12%)	56 (14%)	
Other/Missing	19 (5%)	23 (6%)	22 (6%)	
Smoking history				0.96
Never	77 (19%)	77 (19%)	82 (20%)	
Previous	231 (58%)	227 (56%)	228 (57%)	
Current	92 (23%)	98 (24%)	90 (22%)	
Liver metastases	57 (14%)	53 (13%)	53 (13%)	0.89
Non-squamous histologic subtype				0.81
Adenocarcinoma	377 (94%)	383 (95%)	378 (94%)	
Other	17 (4%)	15 (4%)	19 (5%)	
Unknown or not assessed	6 (2%)	4 (1%)	3 (1%)	
PD-L1 expression #				1
TC0/1/2 and IC0/1	301 (75%)	304 (76%)	299 (75%)	
TC0/1/2 and IC2/3	50 (12%)	51 (13%)	53 (13%)	
TC3 and any IC	49 (12%)	47 (12%)	48 (12%)	
Teff ≥ −1.91 *				0.17
Yes	148 (37%)	177 (44%)	166 (42%)	
No	231 (58%)	210 (52%)	217 (54%)	
Missing	21 (5%)	15 (4%)	17 (4%)	
EGFR mutation status				0.41
Negative	345 (86%)	347 (86%)	352 (88%)	
Positive	45 (11%)	46 (11%)	35 (9%)	
Missing	10 (2%)	9 (2%)	13 (3%)	
Derived neutrophil to lymphocyte ratio				0.097
Median (IQR)	2.2 (1.6–2.9)	2.1 (1.6–3.0)	2.3 (1.7–3.0)	
Missing	10 (2.5%)	4 (1.0%)	8 (2.0%)	
Lactate Dehydrogenase (U/L)				0.7
Median (IQR)	234 (184–318)	233 (185–346)	227 (181–327)	
Missing	17 (4%)	19 (5%)	15 (4%)	
Lung Immune Prognostic Index				0.91
Good LIPI	183 (46%)	188 (47%)	177 (44%)	
Intermediate LIPI	160 (40%)	154 (38%)	165 (41%)	
Poor LIPI	38 (10%)	40 (10%)	43 (11%)	
Missing	19 (5%)	20 (5%)	15 (4%)	

Data are median (IQR) or number of patients (%). P values per Chi-Square test for categorical data and Kruskal-Wallis test for continuous data. # Assessed by immunohistochemical analysis. Tumour cell (TC) or tumour-infiltrating immune cell (IC) group levels (TC PDL-L1 expression on <1% [TC0] or ≥1% to <5% [TC1] or ≥5% to <50% [TC2] or ≥50% [TC3]; IC PDL-L1 expression on <1% [IC0] or ≥1% to <5% [IC1] or ≥5% to <10% [IC2] or ≥10% [IC3]). * effector T-cell gene signature score (Teff; high [≥–1.91] versus low [<–1.91] derived from the average mRNA expression of PD-L1, CXCL9 and IFNϒ relative to a reference gene).

Table S2. Summary of patient characteristics by LIPI group.

Patient Characteristics	**Good LIPI No. 548**	**Intermediate LIPI No. 479**	**Poor LIPI No. 121**	***P***
Treatment arm				0.91
BCP	183 (33%)	160 (33%)	38 (31%)	
ACP	188 (34%)	154 (32%)	40 (33%)	
ABCP	177 (32%)	165 (34%)	43 (36%)	
Age (years)	63 (57–70)	63 (57–68)	63 (58–69)	0.7
Sex				0.65
Male	333 (61%)	288 (60%)	68 (56%)	
Female	215 (39%)	191 (40%)	53 (44%)	
ECOG PS				<0.001
0	268 (49%)	191 (40%)	40 (33%)	
1	275 (50%)	287 (60%)	81 (67%)	
Missing	5 (1%)	1 (<1%)	0 (0%)	

Race				0.67
White	448 (82%)	400 (84%)	95 (79%)	
Asian	72 (13%)	57 (12%)	17 (14%)	
Other/Missing	28 (5%)	22 (5%)	9 (7%)	
Smoking history				0.78
Never	116 (21%)	91 (19%)	22 (18%)	
Previous	310 (57%)	271 (57%)	73 (60%)	
Current	122 (22%)	117 (24%)	26 (21%)	
Liver metastases	48 (9%)	81 (17%)	24 (20%)	<0.001
Non-squamous histologic subtype				0.26
Adenocarcinoma	525 (96%)	450 (94%)	113 (93%)	
Other	19 (3%)	25 (5%)	5 (4%)	
Unknown or not assessed	4 (1%)	4 (1%)	3 (2%)	
PD-L1 expression [#]				0.56
TC0/1/2 and IC0/1	401 (73%)	367 (77%)	94 (78%)	
TC0/1/2 and IC2/3	79 (14%)	56 (12%)	12 (10%)	
TC3 and any IC	68 (12%)	56 (12%)	15 (12%)	
Teff ≥ −1.91 *				0.97
Yes	225 (41%)	198 (41%)	49 (40%)	
No	297 (54%)	263 (55%)	68 (56%)	
Missing	26 (5%)	18 (4%)	4 (3%)	
EGFR mutation status				0.36
Negative	472 (86%)	421 (88%)	106 (88%)	
Positive	65 (12%)	44 (9%)	11 (9%)	
Missing	11 (2%)	14 (3%)	4 (3%)	
Derived neutrophil to lymphocyte ratio	1.9 (1.5–2.3)	2.5 (1.8–3.3)	4.0 (3.4–5.7)	<0.001
Lactate Dehydrogenase (U/L)	193 (166–227)	280 (212–412)	306 (260–445)	<0.001

Data are median (IQR) or number of patients (%). P values per Chi-Square test for categorical data and Kruskal-Wallis test for continuous data. [#] Assessed by immunohistochemical analysis. Tumour cell (TC) or tumour-infiltrating immune cell (IC) group levels (TC PDL-L1 expression on <1% [TC0] or ≥1% to <5% [TC1] or ≥5% to <50% [TC2] or ≥50% [TC3]; IC PDL-L1 expression on <1% [IC0] or ≥1% to <5% [IC1] or ≥5% to <10% [IC2] or ≥10% [IC3]). * effector T-cell gene signature score (Teff; high [≥–1.91] versus low [<–1.91] derived from the average mRNA expression of PD-L1, CXCL9 and IFNϒ relative to a reference gene).

Table 3. Association (adjusted) between LIPI and survival outcomes for patients randomized with ABCP or ACP.

Treatment Arm/Risk Group	OS		PFS	
	**HR [95% CI]**	***P***	**HR [95% CI]**	***P***
ABCP		<0.001		<0.001
Good LIPI (*n* = 163)	1.00		1.00	
Intermediate LIPI (*n* = 153)	2.12 [1.40–3.19]		1.34 [0.99–1.81]	
Poor LIPI (*n* = 40)	7.11 [4.05–12.5]		3.28 [2.11–5.11]	
ACP		<0.001		<0.001
Good LIPI (*n* = 172)	1.00		1.00	
Intermediate LIPI (*n* = 149)	1.71 [1.18–2.48]		1.51 [1.16–1.97]	
Poor LIPI (*n* = 39)	4.81 [2.90–7.97]		2.91 [1.95–4.35]	

CI = confidence interval, HR = hazard ratio, PFS = progression free survival, OS = overall survival; Analyses stratified by sex, PD-L1 expression, and presence of liver metastases; Regression adjusted for age, ECOG PS, race, smoking status, histology, Teff, and EGFR mutation status.
